# Marking can improve defect closure in endoscopic suturing systems

**DOI:** 10.1055/a-2337-9471

**Published:** 2024-06-25

**Authors:** Yoen-Young Chuah, Chung-Ying Lee, Ding-Ek Toh, Hsin-Yu Chen, Kun-Feng Tsai, Kuang-I Fu, Chu-Kuang Chou

**Affiliations:** 1 63292 Division of Gastroenterology and Hepatology, Department of Internal Medicine, Ping Tung Christian Hospital, Pingtung, Taiwan; 2 38000 Department of Nursing, Meiho University, Pingtung, Taiwan; 3 Division of Gastroenterology and Hepatology, Department of Internal Medicine, Taipei Medical University Shuang Ho Hospital, New Taipei, Taiwan; 4 38032 Division of Gastroenterology and Hepatology, Department of Internal Medicine, School of Medicine, College of Medicine, Taipei Medical University, Taipei, Taiwan; 5 38032 TMU Research Center for Digestive Medicine, Taipei Medical University, Taipei, Taiwan; 6 14351 Department of Gastroenterology, Flinders Medical Centre, Adelaide, Australia; 7 60616 Division of Gastroenterology and Hepatology, Cathay General Hospital, Taipei, Taiwan; 8 34903 School of Medicine, College of Medicine, Fu Jen Catholic University, New Taipei, Taiwan; 9 Division of Gastroenterology and Hepatology, An Nan Hospital, China Medical University, Tainan, Taiwan; 10 49048 Department of Medical Sciences Industry, Chang Jung Christian University, Tainan, Taiwan; 11 Department of Endoscopy, Kanma Memorial Hospital, Tochigi, Japan; 12 36597 Division of Gastroenterology and Hepatology, Department of Internal Medicine, Ditmanson Medical Foundation Chia-Yi Christian Hospital, Chiayi, Taiwan; 13 36597 Obesity Center, Ditmanson Medical Foundation Chia-Yi Christian Hospital, Chiayi, Taiwan; 14 36597 Department of Medical Quality, Ditmanson Medical Foundation Chia-Yi Christian Hospital, Chiayi, Taiwan

The emergence of endoscopic suturing systems (ESS) provides a nonsurgical approach to rescuing perforations during endoscopic resection of gastric gastrointestinal tumors 1 2 3 . OverStitch Sx (Boston Scientific, Marlborough, Massachusetts, USA) can be applied to most single-channel endoscopes at the cost of hindering maneuverability. Furthermore, achieving complete full-thickness poses a challenge owing to the anatomic complexities and the obscured visibility of the muscle defect beneath the flap. Endoscopic techniques of ESS remain unfamiliar to most endoscopists. The marking technique is occasionally employed in endoscopic sleeve gastroplasty but has seldom been mentioned in the context of defect closure.

We present the management of a 72-year-old patient undergoing submucosal tunneling endoscopic resection (STER) for a 2-cm gastrointestinal tumor located at the anterior wall of the antrum. After STER, the resulting full-thickness defect was closed using six clips. The patient experienced delayed perforation 3 hours post-procedure, verified with esophagogastroduodenoscopy ( Fig. 1 , Video 1 ). Subsequently, all clips were removed and peritoneal cleansing was performed. We rescued the perforation using the OverStitch Sx device, with the aim of achieving full-thickness approximation.

**Fig. 1:**
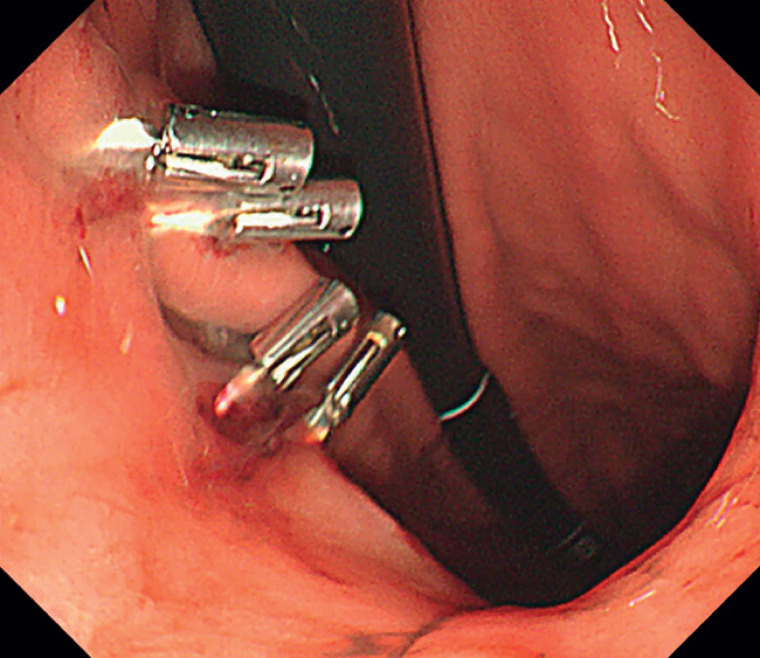
Rupture of the previously clip-closed defect from submucosal tunneling endoscopic resection was noted.

Marking can improve defect closure in endoscopic suturing systems.Video 1

Defect closure presented several challenges. First, targeting the muscle layer was difficult because it was covered by the mucosal flap ( Fig. 2 ). Second, the hindered control and partial loss of vision caused by the suturing device further exacerbated the situation. Prior to suturing, we used Dual J (Olympus, Tokyo, Japan) to mark the areas for stitches ( Fig. 3 , Fig. 4 ). Subsequently, we successfully applied four stitches using OverStitch Sx under the guidance of the marking ( Fig. 5 ). The patient experienced a smooth recovery, progressing to a clear liquid diet within 4 days, and was discharged 6 days post-procedure.

**Fig. 2:**
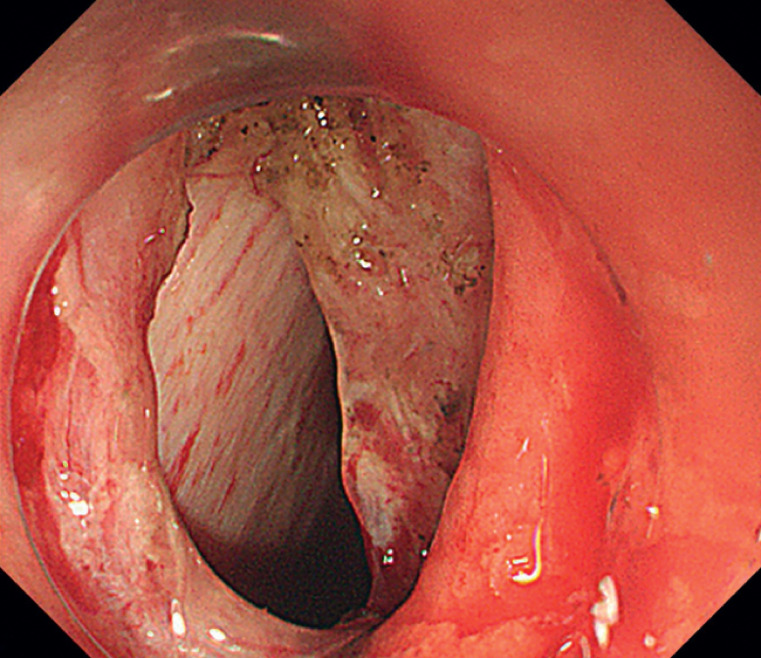
The full-thickness defect was difficult to approximate because it was covered by the mucosal flap.

**Fig. 3:**
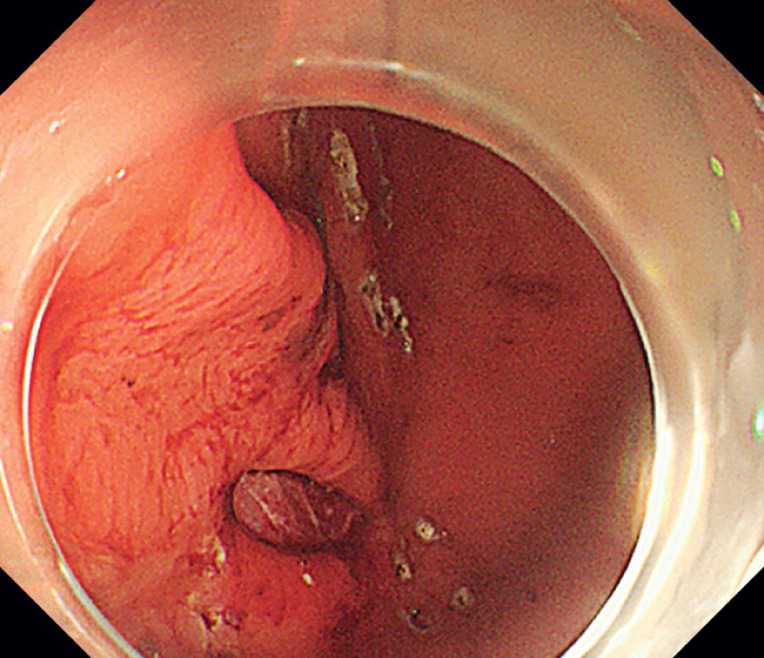
Marking was performed to facilitate seamless stitching during OverStitch Sx suturing (Boston Scientific, Marlborough, Massachusetts, USA).

**Fig. 4:**
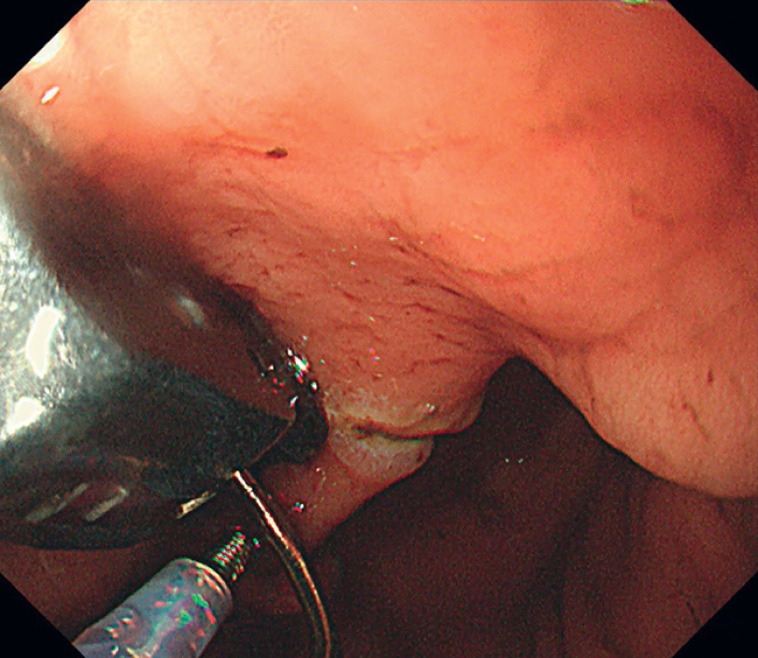
Stitching was performed smoothly following the marking guidance.

**Fig. 5:**
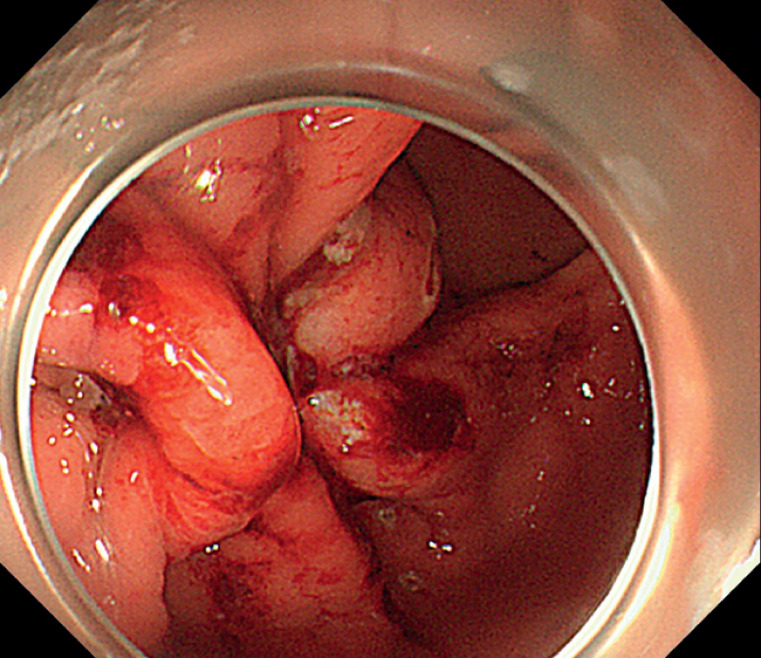
Finally, full-thickness approximation was achieved.

We demonstrate the effectiveness of ESS in managing delayed gastric perforation, which traditionally necessitates surgical intervention. The marking technique can significantly improve the effectiveness of ESS for defect closure.

Endoscopy_UCTN_Code_CPL_1AH_2AZ_3AZ

## Notes

Endoscopy E-Videos https://eref.thieme.de/e-videos
*E-Videos* is an open access online section of the journal *Endoscopy* , reporting on interesting cases and new techniques in gastroenterological endoscopy. All papers include a high-quality video and are published with a Creative Commons CC-BY license. Endoscopy E-Videos qualify for HINARI discounts and waivers and eligibility is automatically checked during the submission process. We grant 100% waivers to articles whose corresponding authors are based in Group A countries and 50% waivers to those who are based in Group B countries as classified by Research4Life (see: https://www.research4life.org/access/eligibility/ ). This section has its own submission website at https://mc.manuscriptcentral.com/e-videos .
